# Use of Mangroves by Lemurs

**DOI:** 10.1007/s10764-016-9905-1

**Published:** 2016-05-14

**Authors:** Charlie J. Gardner

**Affiliations:** 1 Blue Ventures Conservation, 39-41 North Road, London, N7 9DP UK; 2 Durrell Institute of Conservation and Ecology (DICE), School of Anthropology and Conservation, University of Kent, Canterbury, Kent CT2 7NR UK

**Keywords:** Conservation, Madagascar, Primate–habitat interactions, Refuge, Strepsirrhini

## Abstract

Despite an increasing recognition of the ecosystem services provided by mangroves, we know little about their role in maintaining terrestrial biodiversity, including primates. Madagascar’s lemurs are a top global conservation priority, with 94 % of species threatened with extinction, but records of their occurrence in mangroves are scarce. I used a mixed-methods approach to collect published and unpublished observations of lemurs in mangroves: I carried out a systematic literature search and supplemented this with a targeted information request to 1243 researchers, conservation and tourism professionals, and others who may have visited mangroves in Madagascar. I found references to, or observations of, at least 23 species in 5 families using mangroves, representing >20% of lemur species and >50% of species whose distributions include mangrove areas. Lemurs used mangroves for foraging, sleeping, and traveling between terrestrial forest patches, and some were observed as much as 3 km from the nearest permanently dry land. However, most records were anecdotal and thus tell us little about lemur ecology in this habitat. Mangroves are more widely used by lemurs than has previously been recognized and merit greater attention from primate researchers and conservationists in Madagascar.

**Electronic supplementary material:**

The online version of this article (doi:10.1007/s10764-016-9905-1) contains supplementary material, which is available to authorized users.

## Introduction

Mangroves are forests or other vegetated ecosystems that grow in the intertidal areas of subtropical and tropical coastlines around the world. They have attracted increasing conservation attention in recent years, in part as a result of an improved understanding of the ecosystem services they provide, which include carbon sequestration and storage (Donato *et al*. 2011; Nellemann *et al*. 2009; Pendleton *et al*. 2012; Ullman *et al*. 2012), as well as coastal protection and erosion prevention (Alongi 2008; Dahdouh-Guebas *et al*. 2005). In addition, mangroves provide breeding and feeding grounds for a range of marine species (Kathiresan and Bingham 2001; Nagelkerken *et al*. 2008), including fish and crustaceans that sustain major commercial fisheries (Manson *et al*. 2005; Naylor *et al*. 2000), and generate provisioning services for coastal human communities in many countries (Glaser 2003; Rasolofo 1997; van Bochove *et al*. 2014).

Despite the increased recognition of mangrove ecosystem services, our understanding of their importance for the maintenance of terrestrial biodiversity remains patchy (Nagelkerken *et al*. 2008), and this is the case even for charismatic vertebrates such as primates (Nowak 2012). Mangroves are marginal habitats for many terrestrial mammals owing to their extreme and dynamic conditions, including frequent inundation, low botanical and invertebrate diversity, and vegetation that tends to be unpalatable because of its high tannin content (Intachat *et al*. 2005; Kraus *et al*. 2003; Nagelkerken *et al*. 2008; Tomlinson 1995; Vannucci 2001). As a result, there are few obligate mangrove specialists, such as the proboscis monkey (*Nasalis larvatus*), among global primates, though at least 63 further species, including multiple species in the genera *Procolobus*, *Cercopithecus*, *Macaca*, and *Presbytis*, among others, are known to use this habitat facultatively (Nowak 2012): for many, mangroves may be used as a refuge following the loss or degradation of preferred terrestrial habitats. Given that mangroves are among the most threatened of all tropical ecosystems (Duke *et al*. 2007; Valiela *et al*. 2001) and have lost 20%–35% of their global extent since 1980 (FAO 2007; Polidoro *et al*. 2010; Valiela *et al*. 2001), an understanding of their role in maintaining primate populations is essential to inform conservation planning, as well as contributing to our knowledge and understanding of primate–habitat interactions.

**Fig. 1:**
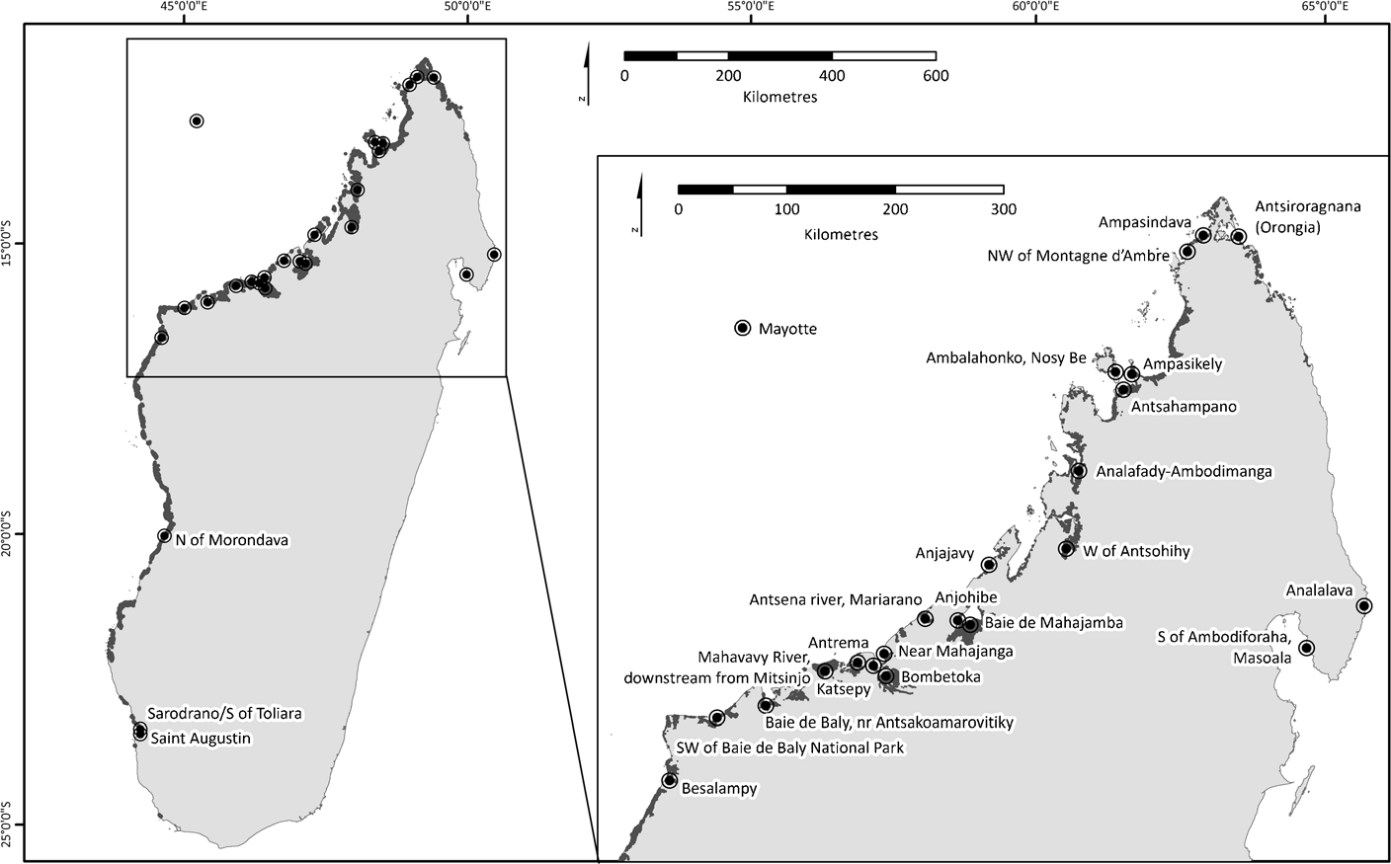
Map of Madagascar showing the distribution of mangroves (dark gray, derived from Giri 2011) and 26 locations at which lemurs have been observed using mangrove habitats.

Madagascar, a global conservation priority boasting unparalleled rates of diversity and endemism among its terrestrial fauna and flora (Brooks *et al*. 2006; Myers *et al*. 2000), is among the countries where mangrove use by terrestrial species is relatively poorly understood. With 213,000 ha of mangroves, Madagascar possesses *ca.* 2% of their global area and is among the top 15 mangrove-rich countries globally (FAO 2007; Giri 2011; Giri *et al*. 2011), yet research into use of the habitat by the country’s reptile, bird, and mammal fauna remains in its infancy. Mangroves are distributed primarily along the west coast, with only small, localized patches in the east (Fig. 1): the greatest coverage is in the northwest, with the largest systems at Mahajamba Bay and Ambaro-Ambanja Bays (Jones *et al*. 2015, 2016). The mangroves are species poor, containing only eight true mangrove species (*Avicennia marina*, *Bruguiera gymnorrhiza*, *Ceriops tagal*, *Rhizophora mucronata*, *Sonneratia alba*, *Xylocarpus granatum*, *Lumnitzera racemosa*, and *Heritiera littoralis*), and little is known about their importance for terrestrial biodiversity: the only group to have been surveyed in mangroves is birds, of which at least 99 species have been recorded (Gardner *et al*. unpublished data). Mangrove ecosystems provide a range of provisioning ecosystem services to adjacent human populations (Rasolofo 1997) and are thus heavily exploited throughout the country. Their management is hampered by a complex legal framework and they are poorly represented in the country’s protected area system; as a result, their extent declined by 21% between 1990 and 2010 (Jones *et al*. 2016).

Among the faunal groups that could be expected to use Madagascar’s mangroves are primates, as Madagascar is among the richest countries for primate diversity, with 105 species, representing >20% of global species-level and 30% of global family-level richness (Mittermeier *et al.*^2013^). However, there remains no evidence of any mangrove specialist lemur species. Until recently our knowledge of mangrove use by lemurs consisted of a few scattered reports; however, two recent reviews have expanded our understanding considerably. Nowak (2012) found reference to four lemur species using mangroves, while Donati *et al*. (2016) collected reports concerning 12 species representing four of the five extant families. Here I expand on the work of these authors with the most thorough and systematic review yet conducted on mangrove use by lemurs. Although published observations of lemurs in mangroves are few, I hypothesized that lemurs may have been observed within this habitat by observers that enter mangroves for reasons other than primate research, and that any such observations may remain unpublished owing to their anecdotal nature. I therefore carried out a mixed-methods review designed to retrieve both published and unpublished reports.

## Methods

To search for published observations, I carried out a systematic literature search for the terms lemur + mangrove and primate + mangrove in relevant online databases and search engines (Academic Search Complete, BioOne, Directory of Open Access Journals, Google Scholar, PrimateLit, Scopus, and Web of Science). I also searched for the term mangrove within the NOE 4D database of articles on natural history in Madagascar (comprising 2852 publications from the period 1658–2008), IUCN Red List web pages for all lemur species, and all volumes of *Lemur News* available in searchable PDF format (volumes 11–18, 2006–2014). To find unpublished observations, I compiled a database of 1243 individuals (including researchers, conservation nongovernmental organization staff, and tour operators and other tourism professionals) who may have spent time in or near mangroves in Madagascar, and sent them a targeted information request by email. Respondents were asked to fill out an online survey or a simple data sheet (both available in English and French; Electronic Supplementary Material) for any observations they had made, and to share the request within their professional networks. I also posted the information request on the Madagascar Environmental Justice Network, an online forum of >1200 members at the time of posting. I collated all the relevant information I retrieved in a database in Microsoft Excel®, but did not perform further analyses because of the opportunistic, i.e., nonsystematic, nature of all observations.

Much of Madagascar’s lemur diversity (particularly among nocturnal genera) is cryptic, preventing accurate field identifications to species level. I tentatively assign observations of such genera to species on the basis of known distributions from Mittermeier *et al*. (2010).

## Results

**Fig. 2:**
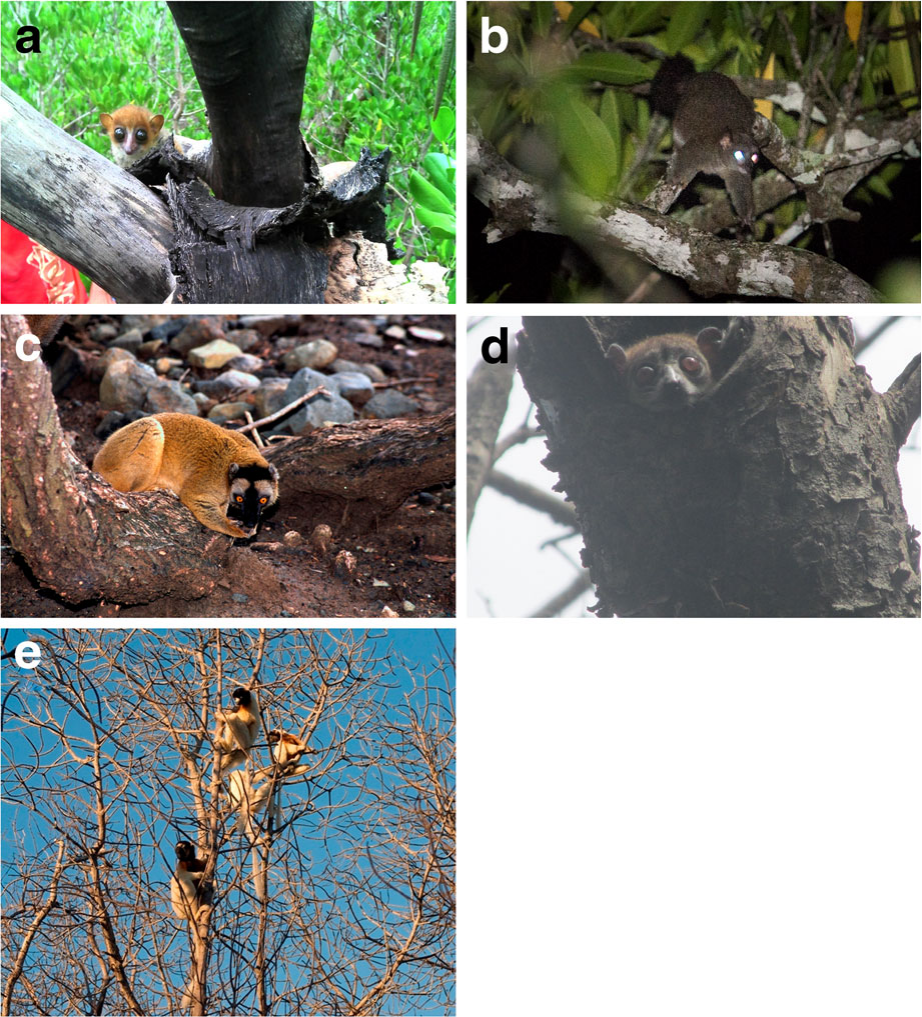
Images of lemurs in mangroves provided by survey respondents. **(a)** *Microcebus* cf. *mamiratra*, disturbed from daytime sleeping site under loose bark of *Ceriops tagal* at Antsahampano (photo: Zo Andriamahenina). **(b)** *Mirza zaza* in *Bruguiera gymnorrhiza* at Antsahampano (photo: Louise Jasper). **(c)** *Eulemur fulvus* eating mud extracted from crab burrows in the mangrove at low tide, southern Mayotte (photo: Laurent Tarnaud). **(d)** *Lepilemur* cf. *grewcockorum* resting in tree hole in *Avicennia marina*, west of Antsohihy (photo: Felix Razafindrajao). **(e)** Group of *Propithecus coronatus* in dead mangrove tree at Antrema (photo: Laurent Tarnaud).

I found references to, or observations of, mangrove use by at least 23 lemur species, representing all five extant lemur families (Cheirogaleidae 7, Lepilemuridae 3, Lemuridae 9, Indriidae 3, and Daubentoniidae 1) (Table **I**; Fig. 1). Of these, 11 species have not previously been reported as using mangrove habitats. The systematic literature search produced peer-reviewed records of mangrove use by two species (*Eulemur fulvus* and *E. macaco*) not reported in previous reviews, as well as a further record of one species (*Lemur catta*) already known to use this habitat. These records may have been previously overlooked because the word mangrove was not mentioned in the title, abstract, or keywords of the papers in question, and so may not have been picked up by search engines. The survey generated responses from 59 individuals including positive reports from 15 respondents relating to observations of 22 species; of these, at least 9 species have not previously been reported from mangrove habitats. Five records were supported by photographs (Fig. 2). Of the records that can be assigned to species on the basis of locality, 20 species are globally threatened with extinction, of which 3 are Vulnerable, 13 Endangered, and 4 Critically Endangered (Schwitzer *et al*. 2013). One additional species was reported by local staff of the Eden Reforestation Project and matches the description of *Cheirogaleus medius*, but I treat this record as unconfirmed because it was reported second hand and thus do not include it in the species totals.

**Table I:** Summary of published and unpublished records of lemurs in mangroves

Species	RL	Location	Observer/source	Details of observation	Month
Family Cheirogaleidae					
*cf. *Cheirogaleus medius*	LC	Southwest of Baie de Baly National Park	Jamie Shattenberg	Based on description provided by local staff working in mangroves, not confirmed	
*Microcebus cf. *danfossi*	EN	Anjajavy	Nick Garbutt	Observed on several occasions	
*Microcebus griseorufus*	LC	Saint Augustin	Donati *et al*. (2016)		
*Microcebus cf. *mamiratra*	CR	Antsahampano	Zo Andriamahenina	Two individuals roosting under loose bark of *Ceriops tagal*	February
*Microcebus mamiratra	CR	Ambalahonko, Nosy Be	Emma Dobinson	1 individual	September
*Microcebus* cf. *myoxinus*	VU	Baie de Baly, near Antsakoamarovitiky	Hawkins *et al*. (1998)	One observed in flowering mangrove tree, probably *Avicennia marina*	
*Microcebus* cf. *myoxinus*	VU	Besalampy	Donati *et al*. (2016)		
*Microcebus cf. ravelobensis	EN	Antsena River, Mariarano	Barry Ferguson	5–10 individuals observed, over 2 nights	January, June
*Microcebus sp.		North of Morondava	Nick Garbutt		
*Microcebus sp.		Analalava	Sebastien Wolf	2 individuals foraging in *Rhizophora mucronata*	June
*Microcebus sp.		Southwest of Baie de Baly National Park	Jamie Shattenberg	Regularly found inside dead mangrove branches (based on testimony of local staff working in mangroves)	
*Mirza zaza	EN	Antsahampano	Charlie Gardner, Louise Jasper	Active in *Bruguiera gymnorrhiza* at night	March
Family Lepilemuridae					
*Lepilemur edwarsi	EN	?	Rasolofo (2011)	No details or reference provided	
*Lepilemur cf. *grewcockorum*	EN	West of Antsohihy	Felix Razafindrajao	3 individuals roosting in tree hole in *Avicennia marina*	February
*Lepilemur tymerlachsoni	CR	Ambalahonko, Nosy Be	Emma Dobinson	2 individuals observed at back of mangrove	August/ September
Family Lemuridae					
*Eulemur albifrons	EN	South of Ambodiforaha	Cortni Borgerson	Single male eating fruit of cf. *Heritiera littoralis*	December
*Eulemur coronatus*	EN	Northwest of Montagne d’Ambre	Donati *et al*. (2016)	Observed at edge of mangroves connecting terrestrial forest, presumed use of mangroves as corridor	
*Eulemur coronatus	EN	Ampasindava	Tojo Razanparany		
*Eulemur coronatus	EN	Antsiroragnana (Orongia)	Razafitsalama Lalao Jeremi	Group of 9 eating flowers of *Sonneratia alba* during dry season	May
*Eulemur flavifrons*	CR	Analafady-Ambodimanga	Dumoulin 2011; Donati *et al*. (2016)	1 male captured in mangrove and 1 group observed traversing rice paddy between mangrove and 2.5-ha forest patch. Author hypothesizes that groups spend most of their time in the mangrove.	
*Eulemur fulvus	NT	Mayotte	Tarnaud and Simmen 2002; Laurent Tarnaud	Groups of 3–6 eating mud extracted from crab burrows at low tide, observed 5–10 times. Also up to 10 licking leaves of mangrove trees in morning, observed 2+ times.	July, August
*Eulemur fulvus	NT	?	Rasolofo (2011)	No details or reference provided	
*Eulemur macaco	VU	Ampasikely	Bayart and Simmen (2005)	Only 1 group out of 3 used mangroves, and in only 1 year out of 3	
*Eulemur macaco	VU	Ambalahonko, Nosy Be	Emma Dobinson	Large group traveling through mangrove, possibly also feeding	March?
*Eulemur mongoz*	CR	Katsepy	Gauthier *et al*. (1999, 2000)	Used as feeding site. Groups of 2 or 3 observed traveling with 9–13 *E. rufus*	
*Eulemur mongoz*	CR	Antrema	Donati *et al*. (2016)		
*Eulemur rufus*	VU	Katsepy	Gauthier *et al*. (1999, 2000)	Used as sleeping site. Groups of 9–13 observed traveling with 2–3 *E. mongoz*	
*Eulemur sanfordi*	EN	NW of Montagne d’Ambre	Donati *et al*. (2016)	Observed at edge of mangroves connecting terrestrial forest, presumed use of mangroves as corridor	
*Lemur catta*	EN	Sarodrano/S of Toliara (several locations)	Donati *et al*. (2016)		
*Lemur catta	EN	Sarodrano/S of Toliara	Sauther *et al*. (2013)	Drinking from freshwater seeps and resting in shade during hottest parts of day	
*Lemur catta	EN	Sarodrano/S of Toliara	Scott *et al*. (ND)		
*Lemur catta	EN	Sarodrano/S of Toliara	Antsa Randrianjohany	Drinking freshwater and eating leaves	May
*Lemur catta	EN	Sarodrano/S of Toliara	Tsibara Mbohoahy	Resting in shade and occasionally eating leaves of *Avicennia marina*	
Family Indriidae					
*Propithecus coquereli*	EN	Anjohibe	Donati *et al*. (2016)		
*Propithecus coquereli*	EN	Baie de Mahajamba	Andriaholinirina *et al*. (2014)		
*Propithecus coquereli*	EN	Anjajavy	Nowak (2012)		
*Propithecus coquereli	EN	Antsena River, Mariarano	Barry Ferguson	2 individuals observed	January, June
*Propithecus coquereli	EN	Anjajavy	Nick Garbutt		
*Propithecus coronatus*	EN	Antrema	Roger and Andrianasolo (2003)	Mangroves are “preferred habitat”	
*Propithecus coronatus	EN	Antrema	Laurent Tarnaud	Five individuals going to roost at night	July
*Propithecus coronatus*	EN	Bombetoka	Nowak (2012)		
*Propithecus coronatus*	EN	Katsepy	Gauthier *et al*. (1999, 2000)	Used as sleeping and foraging sites, sympatric with *Eulemur mongoz* and *E. rufus*	
*Propithecus coronatus	EN	Katsepy	Tojo Razanparany		
*Propithecus coronatus	EN	Katsepy	Rivo Ramanamisata	5 groups, 27 individuals. Mostly resting, sleeping or traveling	December–January
*Propithecus coronatus	EN	Katsepy	Rainer Dolch	Four individuals	
*Propithecus deckenii	EN	Katsepy	Donati *et al*. (2016)		
*Propithecus deckenii	EN	Mahavavy River downstream from Mitsinjo	Nick Garbutt		
Family Daubentoniidae					
*Daubentonia madagascariensis*	EN	Near Mahajanga^a^	Decary 1950		

RL = IUCN Red List status (Schwitzer *et al.* 2013); CR = Critically Endangered; EN = Endangered; VU = Vulnerable; NT = Near Threatened; LC = Least Concern. *Indicates records that have not been reported in previous reviews.

^a^Nowak (2012) refers to a record from Masoala but this is probably erroneous and should refer to the Decary (1950) record.

Neither published records nor survey respondents tended to provide much information with regard to the behavior of observed lemurs within mangrove habitats, at least in part because observations were generally brief, one-off events, and the fact that it may be difficult to ascribe behavior categories to active individuals at night. Nevertheless, the reports indicate that different species may use mangroves for a variety of reasons including shelter, moving between patches of terrestrial habitat, and procuring food or water. In terms of shelter, two nocturnal species, *Microcebus* cf. *mamiratra* and *Lepilemur* cf. *grewcockorum*, have been observed resting or sleeping in mangroves during the day, under the loose bark of *Ceriops tagal* and in a tree hole of *Avicennia marina* respectively (Z. Andriamahanina and F. Razafindrajao *pers. comm*.), while local staff of the Eden Reforestation Project “regularly” find *Microcebus* sp. and another, larger nocturnal species (probably *Cheirogaleus medius*) sleeping in holes and hollow branches of both living and dead mangrove trees, while they are collecting firewood in the mangroves (J. Shattenberg *pers. comm*.). The diurnal *Eulemur rufus* and *Propithecus coronatus* use mangroves as sleeping sites (Gauthier *et al*. 1999, 2000; L. Tarnaud and R. Ramanamisata *pers. comm*.), while *Lemur catta* shelters in the shade of mangroves during the heat of the day (Sauther *et al*. 2013; T. Mbohoahy *pers. comm*.). As well as resting and sleeping sites, mangroves may be used as corridors for travel between patches of terrestrial habitat, e.g., by *Eulemur coronatus*, *E. sanfordi* (Donati *et al*. 2016) and *Propithecus coronatus* (R. Ramanamisata *pers. comm*.).

In terms of foraging and food resources, C. Borgerson (*pers. comm*.) has observed *Eulemur albifrons* eating the fruit of cf. *Heritiera littoralis*, L. Razafitsalama (*pers. comm*.) has observed a group of nine *E. coronatus* eating the flowers of *Sonneratia alba*, and *Lemur catta* occasionally eats the leaves of *Avicennia marina* (T. Mbohoahy *pers. comm*., A. Randrianjohany *pers. comm*.). Mangroves have also been reported as a feeding site for *Propithecus coronatus* and *Eulemur mongoz* (Gauthier *et al*. 1999, 2000; R. Ramanamisata *pers. comm*.), though the species consumed were not specified. Among nocturnal species, S. Wolf (*pers. comm*.) has observed two individuals of *Microcebus* sp. in *Rhizophora mucronata* and Hawkins *et al*. (1998) observed *Microcebus* cf. *myoxinus* in a flowering *Avicennia marina*, although foraging was not directly observed in either case. B. Ferguson has observed 5–10 *Microcebus* cf. *ravelobensis* in mangroves at Mariarano over two nights; although he did not directly observe feeding behavior, the abundance of active mouse lemurs within this habitat suggests that the animals use it for foraging (B. Ferguson *pers. comm*.). On Mayotte (Comoros archipelago) the introduced *Eulemur fulvus* uses mangrove areas to seemingly supplement its diet with minerals; L. Tarnaud has watched groups of 3–6 eating mud extracted from crab burrows at low tide (observed 5–10 times), and up to 10 individuals licking the leaves of mangroves in the early morning (observed 2+ times). In the latter instance, the observer believed that the lemurs may be licking dew as well as salt accreted from the leaves (L. Tarnaud *pers. comm*.). Finally, *Lemur catta* drinks water from freshwater seeps within mangroves in semi-arid areas of far southern Madagascar (Sauther *et al*. 2013; A. Randrianjohany *pers. comm*.).

Among observations for which spatially explicit data were provided (*N* = 21), 81% were of lemurs at the edge of the mangrove or ≤50 m of the nearest permanently dry land. Observations of *Propithecus coquereli* and *Microcebus* cf. *ravelobensis* at Mariarano ranged from 100 m to 1000 m from dry land (B. Ferguson *pers. comm*.), while *Lepilemur* cf. *grewcockorum* and *Mirza zaza* were observed at distances of *ca*. 2 km and 3 km from permanently dry land, respectively (F. Razafindrajao *pers. comm*.; C. Gardner and L. Jasper *unpubl. data*).

Few data are available on the seasonality of mangrove use, though reported observations show no clear patterns in temporal variation. Some species have been reported from mangroves at the same site in both wet and dry seasons, e.g., *Microcebus* cf. *ravelobensis* and *Propithecus coquereli* at Mariarano, and *Propithecus coronatus* at Katsepy, suggesting that mangrove use may be year-round for those species.

## Discussion

Mangroves present a challenging environment for primates as a result of their frequent inundation, low botanical and structural diversity, and foliage that tends to be unpalatable because of a high tannin content (Kraus *et al*. 2003; Tomlinson 1995). They may also harbor lower invertebrate diversity and biomass than terrestrial forests, though comparative data are scarce (Intachat *et al*. 2005; Nagelkerken *et al*. 2008). Nevertheless this review has shown that diverse lemur species are able to use mangroves in some circumstances.

The published and unpublished observations collected here almost double the number of lemur species known to occur in mangroves and, alongside a recent review (Donati *et al*. 2016), increase the known number of global primate species using this habitat by almost 30%, from 64 to 83 (Nowak 2012). They also add a new family (Lepilemuridae) and two new genera (*Lepilemur*, *Mirza*) to the global list. We now know that >20% of lemur species venture into mangroves in at least part of their range, a high percentage given that about half of Madagascar’s lemur species do not have distributions encompassing coastal areas, and almost 40% of species are restricted to eastern regions from which mangroves are largely absent. Based on a visual interpretation of distribution maps (Mittermeier *et al*. 2010), I estimate that 43 lemur species have known ranges likely to encompass mangrove areas, and 53% of these species have now been recorded within them. These findings suggest that the facultative use of mangroves is much more widespread among lemurs than was previously thought, though there remains no evidence that any lemurs are obligate or specialist mangrove dwellers. The lack of specialist mangrove species may be considered surprising given that several lemur species (*Hapalemur* spp., *Prolemur simus*) are adapted to feeding on plants rich in unpalatable chemical components, e.g., bamboos (Poaceae: Glander *et al*. 1989; Yamashita *et al*. 2010), and one (*Hapalemur alaotrensis*) is restricted to aquatic vegetation in a freshwater wetland and may occasionally swim (Petter and Peyriéras 1975; Rendigs *et al*. 2015). Thus neither the unpalatability nor the regular inundation of mangroves need necessarily have constituted a barrier to the evolution of mangrove use by species in these genera.

Lemurs were reported to use mangroves for a variety of reasons, including 1) to rest or sleep in, 2) to rest in the shade during hot parts of the day, 3) to move between patches of forest, 4) to forage on mangrove tree resources (fruit, flowers, leaves), 5) to feed on minerals, and 6) to drink water. Some primarily insectivorous, nocturnal species, e.g. *Microcebus* spp., *Mirza zaza*, may also have been foraging nonvegetal resources, e.g. invertebrates, although foraging was only suspected by the observers and not confirmed. Mangroves may also provide a refuge from predation for some primate species owing to their regular inundation (Matsuda *et al*. 2010; Nowak 2012). Although evidence is lacking, this may also be a factor for some lemurs because nonavian lemur predators, which include Euplerid carnivores, domestic and feral cats and dogs, and a range of snakes (Gardner *et al*. 2015; Goodman 2003; Scheumann *et al*. 2007), are not known to occur in Madagascar’s mangroves. Furthermore, mangroves may provide a refuge from human hunters, who target lemurs through much of Madagascar (Borgerson *et al*. 2016; Gardner and Davies 2014; Golden *et al*. 2014; Razafimanahaka *et al*. 2012).

The extent to which different species use mangroves varies greatly, and some species may occur in this habitat only occasionally or under rare circumstances. For example, Cortni Borgerson (*pers. comm*.) observed *Eulemur albifrons* in a mangrove only once, despite walking through that mangrove regularly over the course of multiple field seasons. Bayart and Simmen (2005) found only one of three focal groups of *Eulemur macaco* at Ampasikely to include mangroves within their territory, and only in one of three years, while Chris Birkinshaw (*pers. comm*.) studied this species in Nosy Be for 18 months without ever observing mangrove use, and villagers in Ankazomborona state that *E. macaco* does not enter mangroves even though it is common in adjacent degraded habitat (C. Gardner *unpubl. data*). Thus mangrove use may occur in some parts of a species’ range but not in others.

For a small number of species mangrove use may be regular behavior, but even then only for a limited population within the species’ ranges. For example, mangroves are said to be the preferred habitat of *Propithecus coronatus* at Antrema (Roger and Andrianasolo 2003), and were reported from there by four respondents in this study, while *P. coquereli* was reported to use mangroves at four different sites. However, most of these species’ ranges lie away from coastal and estuarine areas, and at inland sites the animals are restricted to deciduous dry forests (Andriamasimanana and Cameron 2014; Kun-Rodrigues *et al*. 2014; Rakotonirina *et al*. 2013). Likewise mangrove use by *Lemur catta* has been widely reported from south of Toliara (Donati *et al*. 2016; Sauther *et al*. 2013; Scott *et al*. ND), though this may be the only area within the range of the species in which mangroves occur.

While most observations were made at or close to the edge of mangrove stands this is likely to reflect sampling bias, as their dense growth and regular inundation render mangroves much easier to travel past, on the landward or seaward side, than to travel through. Thus these data should not be regarded as evidence that lemurs tend only to use mangrove edge habitats. Indeed, observations of *Microcebus* cf. *ravelobensis*, *Mirza zaza*, and *Lepilemur* cf. *grewcockorum* at distances of ≥1 km from the nearest dry land demonstrate that these species penetrate deep into mangrove stands. Whereas the former were frequently observed in an area where mangroves are contiguous with intact native forest, the adjacent vegetation at Antsahampano where *Microcebus* cf. *mamiratra* and *Mirza zaza* were observed consisted of coconut plantations and nonnative scrub, while the landscape surrounding the mangrove in which *Lepilemur* cf. *grewcockorum* was observed is entirely deforested. The absence of contiguous native forest cover from these areas suggests that the observed populations are not dependent on source-sink dynamics and the immigration of individuals from areas of higher quality habitat (Pulliam 1988), but are in fact able to maintain viable populations in the mangrove. However, it should not be assumed that these populations will remain viable in the long term because there may be time lags associated with the impacts of landscape deforestation around mangroves, and the remaining lemur populations may thus be carrying an “extinction debt” (Hylander and Ehrlén 2013; Kuussaari *et al*. 2009). It has been hypothesized or demonstrated that lemurs and other primates may use mangroves as a refuge following loss of, or disturbance to, preferred habitats (Galat-Luong and Galat 2005; Gauthier *et al*. 2000; Nowak 2012). Although the presence of lemurs in mangroves lacking adjacent terrestrial habitats may be taken as evidence in support of this hypothesis, we cannot infer that mangroves are suboptimal habitat because we do not know whether these species also used mangroves when connecting terrestrial forests remained.

If mangroves do function as refuge habitats for some nocturnal lemurs, the key resource they provide may be daytime sleeping sites. Most species in the Cheirogaleidae and Lepilemuridae spend the day in nests or tree holes (Mittermeier *et al*. 2010), which provide shelter from predation and assist the maintenance of energy-saving torpor (Dausmann *et al*. 2005; Ganzhorn and Schmid 1998). Respondents in this study reported several species in these families as sleeping within tree holes, under loose bark, and in hollow branches, often from areas lacking alternative sleeping sites, e.g., adjacent to deforested terrestrial landscapes. However, there is some indirect evidence that no lemurs widely use such mangrove refugia. The Madagascar teal (*Anas bernieri*) is a mangrove specialist duck that breeds only in tree holes in mature *Avicennia marina* (Young 2006; Young *et al*. 2013). Suitable nest sites are rare because Madagascar lacks hole-excavating animals such as woodpeckers; thus it has been hypothesized that the teal would not have been able to evolve its breeding habits if it had to compete for tree holes with lemurs (G. Young *pers. comm*.).

Much further research is required to understand better the role of mangroves in the maintenance of lemur populations. This is particularly important for a number of mangrove-using species in northwest Madagascar, such as *Microcebus mamiratra*, *M. danfossi*, *Mirza zaza*, and *Lepilemur grewcockorum*, which are classed as Endangered or Critically Endangered on the basis of their small range and declining area of occupancy (AOO). However, even if mangroves are demonstrated to provide important habitat for these species they may not be more secure than terrestrial forests, as mangrove deforestation rates may match or even exceed those of terrestrial forests in some parts of the region (Jones *et al*. 2014, 2015; ONE *et al*. 2013).

## Conclusions

The mixed-methods approach I adopted for this review allowed the collection of numerous published and unpublished observations that together have greatly expanded our knowledge of mangrove use by lemurs. We now know that more than half of all lemurs with distributions encompassing mangrove areas are able to use them facultatively in some circumstances, and may do so for a number of reasons. However, observations are almost entirely anecdotal so our understanding of the role of mangroves in the maintenance of lemur populations remains extremely limited. Improving our knowledge will require systematic surveys of the country’s remaining mangroves to understand better which species occur in them and where, as well as comparative focal studies of lemur populations in mangroves and adjacent terrestrial habitats to understand better the ecological role of mangroves in the maintenance of populations. Given the difficulties of surveying mangroves, camera trap and video technologies may provide useful tools in this regard. Ninety-four percent of all lemur species are threatened with extinction, primarily as a result of ongoing habitat loss (Schwitzer *et al*. 2013), and conservation efforts are focused overwhelmingly on Madagascar’s terrestrial forests on which the vast majority of the country’s lemurs depend. This review suggests that mangroves may provide important refuges and other resources for some species, and thus that Madagascar’s mangroves merit increased attention from the country’s primatologists and lemur conservationists.

## Electronic supplementary material

Below is the link to the electronic supplementary material.

ESM 1 (DOC 42 kb)
