# Supplementary material for: Use of Mangroves by Lemurs
Source: Int J Primatol. 2016 May 14;37:317–32. doi: 10.1007/s10764-016-9905-1 (PMC4978774; doi:10.1007/s10764-016-9905-1)
Supplement: Supplementary file 1 — (DOC 42 kb) [file 10764_2016_9905_MOESM1_ESM.doc]

**Electronic Supplementary Material**

**Use of Mangroves by Lemurs**

**Charlie J. Gardner**

**Data Sheet: Lemurs in Mangroves**

Name:

Date of observation:

Species observed (or genus if not known):

Location (with coordinates):

Number of lemurs observed (and description of group composition):

Description of observation: What was the lemur doing? Please provide as much detail as possible:

Mangrove tree species in which lemurs were observed (local Malagasy or scientific name):

Description of mangrove habitat where observation was made:

Approximate distance to nearest permanently dry land:

Was the mangrove forest contiguous with native forest/thicket? Please describe the adjacent vegetation:

Other observers present:

Any additional information:

**Fiche de Données: L’utilisation des mangroves par les lémuriens**

Nom:

Date de l’observation:

Espèce observée (ou genre si pas identifiée):

Lieu (avec géocoordonnées):

Nombre de lémuriens observées (et description du group):

Description de l’observation: Que faisait le lémurien? Merci de donner autant de détails que possible:

Espèce de mangrove dans lequel vous avez vu le lémurien (nom local ou scientifique):

Description de la mangrove ou le lémurien était observé:

Distance jusqu’à la terre ferme:

Etait la mangrove contiguë avec de la forêt (ou fourre) terrestre? Merci de décrire la végétation terrestre adjacente:

Autres témoins présentes:

Autres informations:
